# Navigating the maze of requirements for obtaining approval of non-interventional studies (NIS) in the European Union

**DOI:** 10.3205/000225

**Published:** 2015-11-17

**Authors:** Isabelle Ramirez

**Affiliations:** 1 Zerimar Consulting, Munich, Germany

**Keywords:** non-interventional studies, observational studies, ethics committee, competent authorities, data protection authorities, regulatory approval

## Abstract

**Objective:** The purpose of this article is to give an overview of the complexities and unexpected regulatory requirements for obtaining approval of multinational and multicentre non-interventional studies (NIS) in the European Union (EU).

**Methods:** The websites of national competent authorities (CAs), ethics committees (ECs) and data protection (DP) authorities were consulted to find regulations and guidance information related to the authorisation of NIS in various member states of the EU.

**Results:** Many additional hurdles, neither disclosed nor clear in the various regulations/guidances for NIS, were identified. Although approval from the CA is not needed for NIS, in many countries request of CA opinion is nevertheless recommended, prior to submission to the EC, to obtain confirmation that the planned NIS does not fall in the interventional trial category. Clinical trial insurance was required in some countries. In countries like Belgium and Italy, the multicentre NIS required the approval from a central EC and local ECs as a single central EC opinion was not considered sufficient. The EC document requirements for submission and the fees were extremely variable among all member states. Additional approvals from data protection authorities and insurance companies were required in some countries.

**Conclusions:** The process of obtaining approval for multicentre and multinational NIS is time consuming due to lack of transparency and the different regulatory requirements among member states. The EU pharmacovigilance legislation and clinical trial regulation No 536/2014 is a step forward in providing a regulatory framework for PASS (post-authorisation safety studies) and low intervention clinical trials, but since regulation No 536/2014 excludes NIS, it will be difficult to enforce harmonization of requirements for approval of NIS among member states.

## Zusammenfassung

**Ziel:** Dieser Artikel soll über die Komplexität und unerwartete regulatorische Anforderungen bei multinationalen und multizentrischen, nicht-interventionellen Studien (NIS) in der Europäischen Union aufklären.

**Methoden:** Die Internetseiten der zuständigen nationalen Behörden, Ethikkommissionen (EK) und Datenschutzbehörden wurden konsultiert, um Vorschriften und Richtlinien zum Genehmigungsverfahren von NIS in den verschiedenen Mitgliedstaaten der EU zu finden.

**Ergebnis:** Viele zusätzliche Hürden wurden identifiziert, die weder offengelegt noch deutlich in den jeweiligen Verordnungen/Richtlinien für NIS beschrieben waren. Obwohl die Genehmigung durch die nationale Behörde für NIS grundsätzlich nicht erforderlich ist, wird in vielen Ländern dennoch empfohlen die geplante NIS – vor dem EK-Antrag – der nationalen Behörde anzuzeigen, um die Bestätigung zu erhalten, dass die geplante NIS nicht in die Kategorie „interventionelle Studie“ fällt. Zudem ist in einigen Ländern eine Probandenversicherung erforderlich. In vielen Ländern, in denen multizentrische NIS geplant sind, bedarf es zusätzlich zur Zustimmung der zentralen Ethikkommission auch noch die zustimmende Bewertung aller lokalen Ethikkommissionen, denn ein zentrales EK-Gutachten wird nicht als ausreichend betrachtet. Die Anforderungen an EK-Dokumente und an Gebühren für die Einreichung variieren stark unter allen Mitgliedsstaaten. Zusätzliche Genehmigungen von den Datenschutzbehörden und Versicherungsgesellschaften sind in einigen Ländern einzuholen.

**Schlussfolgerung:** Das Genehmigungsverfahren für multizentrische und multinationale NIS ist zeitaufwendig, vor allem wegen des Mangels an Transparenz und den unterschiedlichen regulatorischen Anforderungen der Mitgliedsstaaten. Die EU-Rechtsvorschriften zur Pharmakovigilanz und die neue EU-Verordnung Nr. 536/2014 über klinische Studien sind zwar ein Schritt nach vorn bei der Schaffung eines Rechtsrahmens für PASS (post-authorisation safety studies) und minimalinterventionelle klinischen Studien, aber da die Verordnung Nr. 536/2014 nichtinterventionelle Studien ausschließt, wird es schwierig sein, eine Harmonisierung der Anforderungen für die Genehmigung von NIS zwischen den Mitgliedsstaaten durchzusetzen.

## Introduction

The European Clinical Directive 2001/20/EC [1] includes a definition of non-interventional studies (NIS) (see Table 1 (Tab. 1)). However, the directive itself and the recent clinical trial EC regulation No 536/2014 repealing 2001/20/EC [2] and other clinical trial related guidelines do not include details on the complete legal framework and requirements to obtain regulatory approval for such studies in each member state of the European Union (EU).

Non-interventional studies are in many European countries still referred as observational studies. Typical examples of NIS are: registry studies (patient or disease registry), phase IV studies (post-authorisation safety studies (PASS)), post-marketing studies, among others. Because NIS include post-marketing studies and such type of studies were misused by pharmaceutical companies to increase marketing sales of approved medicinal products [3] and NIS were frequently scientifically poorly designed [4], country self-regulatory bodies e.g. pharmaceutical associations have issued codes of conduct to properly design and conduct such studies in each country of the EU. As a consequence, a patchwork of regulations and codes of conducts have to be followed in multinational NIS in Europe. 

Because the requirements in terms of the contents of the NIS application dossier is usually much less than for an interventional study, the administrative burden of obtaining approvals of NIS in Europe is frequently underestimated by commercial and non-commercial sponsors of such studies as pointed out in several publications [5], [6]. The purpose of this article is to give an overview of the complexities and unexpected regulatory requirements that might jeopardize the prompt approval and study initiation of multinational and multicentre NIS in the EU. 

## Methods

The author was commissioned to obtain the required approvals for the conduction of two NIS:

1. a retrospective and cross-sectional study in adult patients suffering a neurologic disease; and
2. a prospective study with a recently approved medicinal product in the same target population.

The first NIS aimed to find out the current therapies in the pursued indication in the selected member states (use of resources, cost, etc.) and patient quality of life (QoL) while the second NIS aimed to collect the same information, but in terms of a specific therapy approved recently in the EU for the same indication. Both studies were voluntary and not imposed by a competent authority (CA). 

The national CA, local ethical committees’ (EC) and data protection (DP) authorities’ websites were consulted to find the regulations and guidance information related authorisation of NIS in various member states of the EU. The selected countries were: Austria, Belgium, Czech Republic, Finland, France, Ireland, Italy, The Netherlands, Norway, Poland, Portugal, Slovakia, and Sweden. 

The experience accumulated during this period is summarized in terms of additional administrative hurdles neither disclosed nor clear at first glance in European, national directives/regulations or guidelines.

## Results

### Hurdle No. 1: Is my study really an NIS?

Although in all participating countries of the planned studies the approval from the national CA was not required (as expected), it is nevertheless advisable in e.g. Finland and Denmark to contact the CA prior to ethical committee submission, in order to obtain CA confirmation that the planned study is indeed non-interventional. This is recommended because the non-interventional protocol as designed by sponsors might be interpreted by a CA as interventional, and therefore the study in this country will require CA approval as an interventional trial. In Denmark, for example, the CA was contacted and after a series of communications, which included a detailed analysis of the protocol and clarification of questions, the CA issued an official letter stating that the study fell in the NIS category. The same study protocol was interpreted, however, as interventional by the Finnish authorities due mainly to the QoL patient questionnaire which was considered by the CA as not falling within routine practice. 

The retrospective/cross-sectional study contained a QoL questionnaire for the patients to assess how they felt about the treatment they received for their disease in the past (last 12 months). The selected central EC in Belgium interpreted this questionnaire as prospective and not as retrospective or cross-sectional. Consultation with the CA did not persuade the EC that the study was indeed retrospective/cross-sectional with the consequence that the title of the study protocol was modified in this country to suit the opinion of the consulted EC. In countries like the Netherlands, a questionnaire could be considered as an intervention (and therefore the study is no longer an NIS) if the questionnaire is very long or highly personal. Consequently prior clarification with the Centrale Commissie Mensgebonden Onderzoek (CCMO; CA in this country) is advisable in the Netherlands as well. 

Table 1 (Tab. 1) summarizes each requirement for NIS as stipulated in the current clinical trial legislation and how each requirement could be interpreted by CAs and ECs and some important considerations when writing an NIS protocol. As the requirements to fulfill the criteria for NIS are cumulative (each condition must be fulfilled) it is crucial to design the study protocol and e.g. patient questionnaires to comply with each requirement. For example, compulsory diagnostic test must be standard among all member states where the study takes place and those tests which are not standard in all participating countries, can be included in the protocol as optional and to be conducted only if routine in the country in question. 

### Hurdle No. 2: Do I need clinical trial insurance to cover for an NIS?

In general the answer is no, but in Belgium, clinical trial insurance was required and obligatory by law to cover a potential unauthorised access of patient data by non authorised personnel, in spite of the presence of several sets of IT safety measures to protect the patient’s real identity by for example collecting data under a code number not related to the patient’s initials or birth date (process known as key-coding the trial subject identity). 

The EU Regulation No 536/2014 repealing 2001/20/EC [2] states indirectly that for the so called low risk clinical trials (see Table 2 (Tab. 2)), the insurance coverage of the principal investigator (medical practitioner), the institution, or a product liability insurance should be enough, and no extra clinical trial insurance should be requested by ECs, unless there are additional risks associated with the study itself. In the future, a clear set of rules with respect to insurance should be implemented in each member state in order to bring some harmonization among member states in the EU with respect to this requirement. 

### Hurdle No. 3: Is a single EC opinion per country enough?

For interventional studies, a single opinion is emitted by the central EC on behalf of all local ECs, but still local EC approvals are required in case of multicentric studies as the local ECs have the responsibility to evaluate that the personnel of the local clinical site are qualified and that the resources are available to conduct the study. In Italy and Belgium where the approval for interventional trials is obtained from the central and the local ECs, the same time consuming preparation process of collecting documents for the application dossier applies to NIS. Therefore in these countries, the time investment required to prepare the application dossier for an NIS is the same as for an interventional study. In countries like Austria, Norway and Poland, however, only one EC approval is required for multicenter studies as per local code of conduct. In France, NIS are not evaluated by the ECs (Comité de protection des personnes; CPP), but by the “Comité consultative sur le traitement de l’information en matière de recherché” (CCTIRS). 

In Austria and Denmark, ethical approval is not required, but since many international journals will reject publishing NIS data for which no EC positive opinion is available, it is recommended to request the approval of at least one EC. In Denmark, as no EC will evaluate an NIS protocol, to circumvent the problem of publication, the ECs in Denmark are willing to write a letter to journal publishers to explain that EC assessment is not performed and should not be expected for NIS conducted in this country. 

The time to evaluate the application dossier by the ECs for NIS is not regulated as for interventional studies, and in our case the approval times varied between 1 month to 3 months.

### Hurdle No. 4: Are the EC requirements among member states the same or different?

The core set of documents required by the EC is almost constant across Europe (see Table 3 (Tab. 3) – left column). In some countries some additional documents are required (Table 3 (Tab. 3) – right column). The applicant must be prepared to respond to some critical questions contained in the application form for an NIS e.g. risk/benefit, data protection, etc. For some countries, like the Netherlands, Ireland and Norway, the applicant must be the principal investigator (PI) and not the commercial sponsor or a contract research organization (CRO). 

As each country has its own set of documentation requirements and changes occurs from year to year, applicants of NIS must consult the ECs websites for the specific set of documents required at the time of submission. The European Network of Research Ethics Committees (EUREC) provides links to RECs for all member states in the European Union (http://www.eurecnet.org/index.html). The above table is a good start for planning resources to support a NIS. Translation of the protocol synopsis, questionnaires (if applicable) and patient information and informed consent form into the country language(s) is required in all member states. 

### Hurdle No. 5: What are the EC fees for the required evaluations?

Although the documentation requirement for NIS is not as large and complex as that for interventional studies, many countries do not make a distinction and charges large fees for evaluation of NIS. Table 4 (Tab. 4) shows examples of fees at the time of the application.

In countries like Italy, fees of around € 2,000 (variable depending of the ECs from €800 to €4,000) are payable to the central and to each local EC. In Italy, the fee is the same regardless of whether the EC issues a single opinion on behalf of all ECs (so called *parere unico*) or just a local opinion. 

### Hurdle No. 6: Do I need other approvals besides obtaining a positive EC opinion?

Depending on the design of the study, approval from or notification to data protection (DP) authorities is required. In some member states the responsibility to assess the design of the study in terms of data protection are clearly delegated to the ECs of the country and therefore the DP authorities in these countries are not involved in approving NIS. In countries like Denmark, if the data controller (the organization assigned to collect and process the data) is located outside the respective country, no declaration is needed to the country’s data protection authority. In a few countries, namely France, Portugal and Belgium, an application is required for approval from the DP authority regardless of where the data controller is located and regardless of whether the data from patients are pseudonymised or key-coded. The DP approval process in these countries was long (2 months for Belgium, 2.5 months France, 8 months Portugal) and delayed study initiation even though all other approvals were already obtained. Therefore, it is advisable to place a request to the country DP authorities if notification or approval is required by describing the data process flow and the location of the responsible for data collection and the planned data processing. It is also advisable to place such request for approval from the DP authorities in parallel with the EC submissions in order to save time.

Another additional voluntarily requisite stated mainly in the various codes of conducts (e.g. European Network of Centres for Pharmacoepidemiology and Pharmacovigilance (ENCePP) and member states codes of conduct) and scientific journals is to publish the study results in an European clinical trial database [7]. In countries like Austria and Slovakia the study must be also registered in a country specific local database for NIS. Besides this registration of the NIS in a database, notification to the CA is compulsory in some member states and in particular for post-authorisation safety studies (PASS), which are imposed by a CA as described in annex to GVP module VIII – Table VIII Add I.1 [8]. For PASS initiated voluntarily by a pharmaceutical company, the registration in a EU database such as ENCePP is recommended in addition to notification to CA as defined in the annex to GVP module VIII-table VIII Add.I.2 [8].

If the sponsor pharmaceutical company is registered as member of a country pharmaceutical association, many of these associations require notification of the study after approval from the ECs and other agencies (as applicable).

Slovakia includes in their process, a very specific requirement, which is the authorisation of all major insurance companies in a country before the study can be initiated. Only those patients whose insurance company has approved the NIS can be included in the study.

After approval of the study, some member states require the notification of non-substantial and substantial amendments of study protocol and informed consent form, and all member states require the sending of the study report or publication to one or several authorities after study completion. 

## Conclusion

The biggest challenge in terms of regulatory compliance of NIS in the EU is to find out the complete set of required approvals and notifications to start an NIS in each of the member states of the EU. Not only positive opinions from one or more ethics committees, but also approval or notification from or to data protection authorities and other entities e.g. insurance companies might be required depending on the country.

There is a lack of country guidelines for NIS covering the complete set of requirements for approval, notification and registration of an NIS. One exception is Austria, where the BASG/AGES (Bundesamt für Sicherheit im Gesundheitswesen) has published a guideline [9] containing updated and detailed description of the complete pathway for approvals, notification and registration of NIS. The BASG guideline, in addition, explains in detail the obligations and duties of sponsors after study approval and database registration in terms of amendments, report of adverse events, and where and when to send the final study report after study completion, etc. Such complete guidelines are very useful, but unfortunately very rare in almost all EU countries. 

In every country included in the retrospective and prospective planned studies, challenges were faced because the EC evaluation of the protocol and additional documents took as long as for an interventional trial. In countries where approval from data protection authorities was required, the DP authorities were very slow (2–8 months) in evaluating the study with consequent delays in study initiations.

For PASS, the current pharmacovigilance legislation [10] and concomitant guidelines [11] have helped to establish a clearer legal framework and requirements for approval of such studies. Also the ENCePP platform with its code of conduct, checklist for requirements, etc. has helped to bring some clarification and harmonization, but only from the perspective of the CA. 

The EU regulation No 536/2014 repealing 2001/20/EC [2] adds a new category of clinical trial called low intervention clinical trial (see Table 2 (Tab. 2)) which includes the condition, among others, of allowing the use of additional diagnostic and monitoring procedures if they pose minimal risk or burden to the patient (see condition (c) in Table 2 (Tab. 2)). However, the regulatory framework for conducting an NIS in Europe will still remain ambiguous as this regulation does not apply to NIS and its definition remains unclear because NIS will be any type of study that does not fall into the category of clinical trial (see (4) in Table 2 (Tab. 2)). 

The Regulation No 536/2014, which will apply not earlier than 28 May 2016, is a step forward in terms of which conditions should be fulfilled for a trial to be a low intervention trial. However, the regulation will increase the regulatory hurdles for such studies as it will require approval from the CA of the member states where the study will take place, in addition to the required positive opinion from the ECs (not specified whether one or several ECs) and approvals from other entities such as DP authorities. Because regulation No 536/2014 regulates only low intervention trials from the perspective of the approval from member states, the required approvals from ECs and DP authorities for NIS will remain an area of uncertainty and variability among the member states in the EU. Consequently, simplification and harmonization of the process required among EU member states to obtain regulatory approval for low intervention and non-interventional studies will remain on the wish list of sponsors of NIS for the foreseeable future. 

## Notes

### Competing interests

The author declares that she has no competing interests.

### Acknowledgements

The author would like to thank Dr. William Blackley and Angelika Stobbelaar for the lingustic review of this manuscript.

## Figures and Tables

**Table 1:**
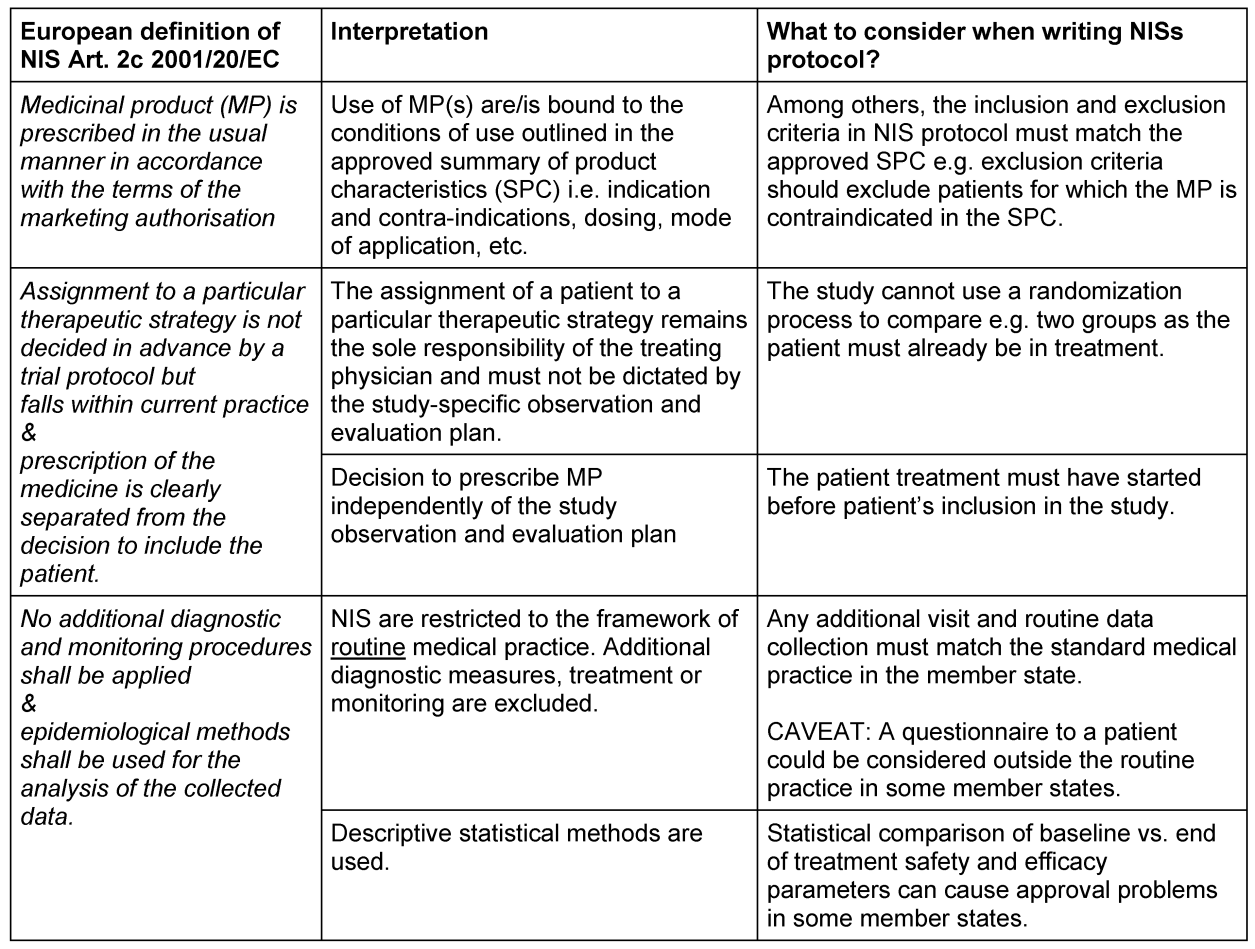
NIS definition and national interpretations

**Table 2:**
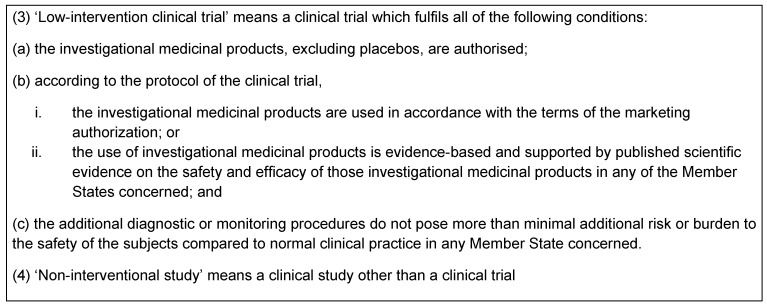
Regulation No 536/2014 – Definition of low intervention trial and ‘non-interventional study’

**Table 3:**
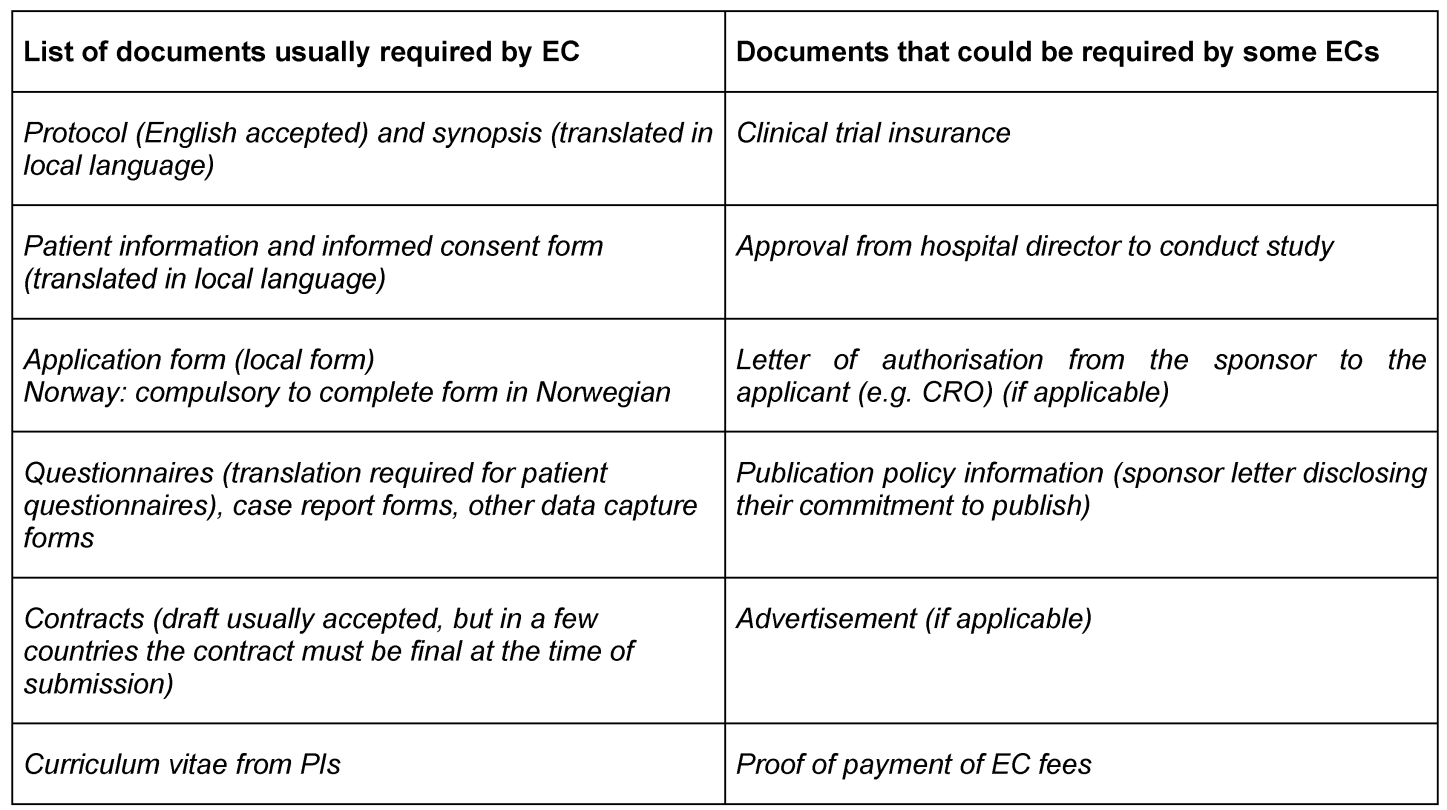
Documents required by ECs across the EU

**Table 4:**
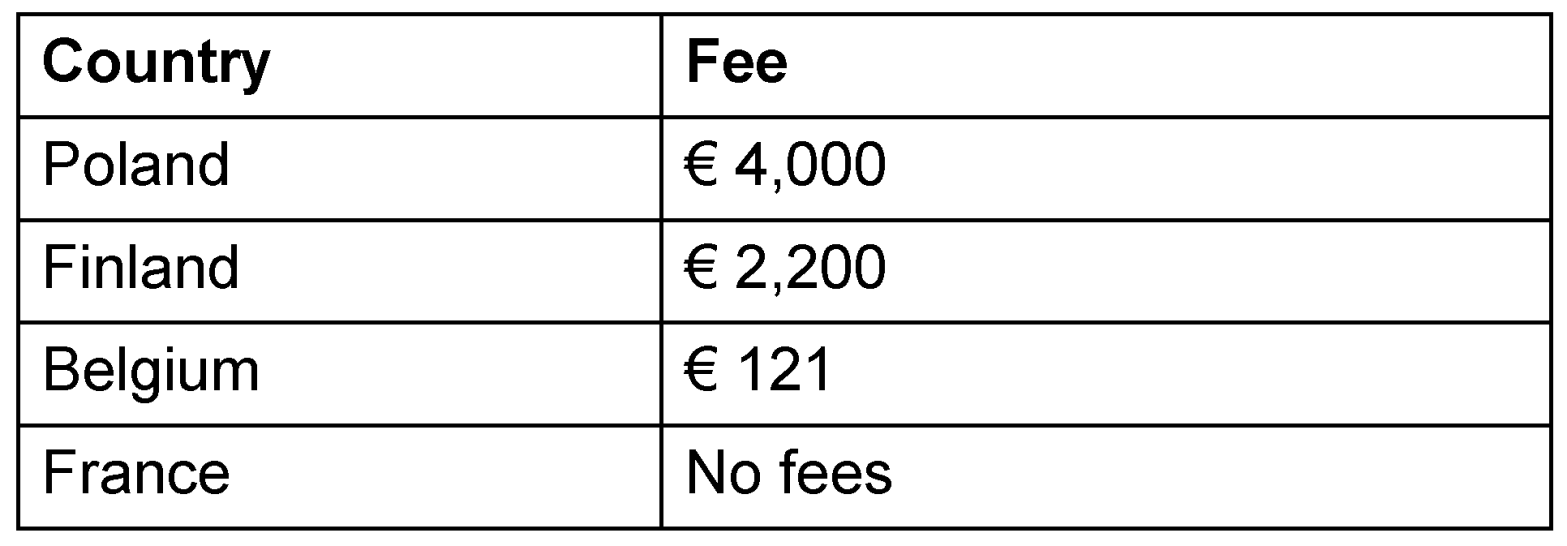
Fees to be paid in some member states of the EU
